# Effect of Pre-Setting Exposure to 17% EDTA on Physicochemical and Surface Properties of AH Plus Bioceramic and AH Plus Endodontic Sealers

**DOI:** 10.3390/dj14040191

**Published:** 2026-03-24

**Authors:** Gerardo Alberto Salvador Gomez-Lara, Carlos Alberto Luna-Lara, Rogelio Oliver-Parra, Suria Sarahi Oliver-Parra, Carlos Roberto Luna-Dominguez, Jorge Humberto Luna-Dominguez

**Affiliations:** Faculty of Dentistry, Autonomous University of Tamaulipas, Av. Universidad Esq. con Blvd, Adolfo Lopez Mateos S/N, Tampico C.P. 89337, Tamaulipas, Mexico; gerardo.gomez@docentes.uat.edu.mx (G.A.S.G.-L.); roliver@docentes.uat.edu.mx (R.O.-P.); suria.oliver@uat.edu.mx (S.S.O.-P.); cldominguez@docentes.uat.edu.mx (C.R.L.-D.); jhluna@docentes.uat.edu.mx (J.H.L.-D.)

**Keywords:** endodontic sealer, calcium silicate–based sealer, epoxy resin sealer, ethylenediaminetetraacetic acid (EDTA), irrigation residues

## Abstract

**Background/Objectives**: Residual EDTA may persist after smear-layer removal and after the application of contact sealers during setting. This in vitro study compared the effect of 1 min of pre-setting surface contact with 17% EDTA (vs. distilled water) on a calcium silicate-based sealer (AH Plus Bioceramic Sealer) and an epoxy resin-based sealer (AH Plus). **Methods**: Discs were prepared (*N* = 108) in a 2 × 2 design (*n* = 27/group); per group; *n* = 12 were used for solubility followed by eluate pH using the same specimens/eluates after 24 h immersion in distilled water; *n* = 12 were used to test Vickers microhardness on an independent set after setting; and *n* = 3 were used for SEM/EDS. **Results**: Data were analyzed at α = 0.05 using Kruskal–Wallis tests followed by Bonferroni-adjusted pairwise Mann–Whitney U tests for solubility and eluate pH, and one-way ANOVA was performed followed by Tukey’s post hoc test to assess the microhardness. Solubility differed among groups (*p* < 0.001) and was higher for the bioceramic sealer than for the resin sealer; pre-setting EDTA exposure increased solubility for the AH Plus Bioceramic Sealer (0.86 ± 0.08% to 1.30 ± 0.16%) and decreased solubility for AH Plus (0.34 ± 0.04% to 0.22 ± 0.03%) (*p* < 0.05). The eluate pH also differed among groups (*p* = 0.001) and was higher for the bioceramic sealer (≈11.7) than for the resin sealer (≈8.7–9.3), with no within-material differences (*p* = 0.999 and *p* = 0.851). Microhardness differed among groups (*p* < 0.001) and was higher for AH Plus (239.70–246.92 HV) than for AH Plus Bioceramic (131.72–170.83 HV); EDTA reduced microhardness only for the bioceramic sealer (*p* < 0.001), with no significant change for AH Plus (*p* = 0.475). Descriptive SEM/EDS findings suggested increased surface irregularities and lower surface Ca for AH Plus Bioceramic after EDTA exposure (12.68 to 7.31 wt%). **Conclusions**: Pre-setting EDTA contact therefore produced material-dependent changes in early properties and adverse surface-related effects in the calcium silicate-based sealer, supporting thorough chelator removal before obturation.

## 1. Introduction

Endodontic sealers are essential to root canal obturation because they occupy the interface between the core material and the canal walls, address discrepancies and anatomical irregularities that are not occupied by the core material, and help to establish a continuous three-dimensional seal that limits microbial leakage and supports long-term treatment success [1,2,3]. In clinical service, this interfacial layer is exposed to fluids and chemical residues; therefore, its dimensional stability and resistance to dissolution are clinically relevant. Any degradation of the sealer phase may create interfacial discontinuities that facilitate coronal or apical leakage and reinfection [2,3]. For this reason, understanding how contemporary sealers behave when challenged by clinically realistic chemical conditions remains a priority in endodontic materials research.

The chemical environment at the time of obturation is strongly influenced by chemomechanical preparation and irrigation. Instrumentation unavoidably generates a smear layer composed of organic and inorganic debris that can occlude dentinal tubules and reduce the penetration of irrigants, medicaments, and sealers [4]. Current irrigation protocols therefore rely on solutions with complementary actions. Sodium hypochlorite (NaOCl) is widely used for organic tissue dissolution and antimicrobial control, chelating agents such as 17% ethylenediaminetetraacetic acid (EDTA) target the inorganic component of the smear layer, and adjunctive solutions such as chlorhexidine have been used when antimicrobial substantivity is desired despite lacking tissue-dissolving capacity [5,6,7,8]. The clinical value of EDTA lies in smear-layer removal and dentin surface conditioning before obturation. Classic SEM-based studies have shown that sodium hypochlorite alone does not remove the smear layer, whereas protocols combining sodium hypochlorite with EDTA or EDTAC result in significantly cleaner canal walls [9,10]. In addition, the final irrigation sequence influences the subsequent interaction of resin-based sealers with dentin, including sealer penetration and adhesion, further underscoring the clinical relevance of the chelating step before obturation [11,12]. Although EDTA is effective, complete removal from the root canal system is difficult because of anatomical complexity and the potential for solution retention within isthmuses, lateral anatomy, and dentinal tubules [8]. Residual EDTA can persist at the time of obturation and may alter the chemical conditions at the sealer–dentin interface, with potential consequences for setting reactions, surface stability, and long-term interfacial integrity [8,11,12].

Epoxy resin-based sealers have traditionally been selected because of their favorable handling, low solubility, and good dimensional stability. AH Plus (Dentsply DeTrey, Konstanz, Germany) is widely considered a benchmark material, which is supported by evidence of physicochemical stability and satisfactory sealing performance [2,13]. AH Plus mainly sets through epoxy polymerization and forms a relatively stable hydrophobic matrix [2,13]. However, epoxy resin sealers are not designed to provide bioactive effects such as sustained ion release or interfacial mineral deposition [14,15]. These limitations have contributed to an increasing interest in hydraulic calcium silicate-based sealers, often referred to as bioceramic sealers, which are intended to interact with dentin and surrounding tissues through hydration chemistry and bioactive surface reactions [14,15,16,17].

Hydraulic calcium silicate sealers set through hydration reactions that form calcium silicate hydrate and promote the release of calcium and hydroxyl ions. This chemical behavior can create an alkaline environment and may support the precipitation of apatite-like phases under suitable conditions [15,16,17,18]. These mechanisms have been associated with bioactivity, favorable biological responses, and interfacial mineral deposition that may enhance adaptation and sealing over time [15,18,19]. AH Plus Bioceramic Sealer (Dentsply Sirona) is a calcium silicate-based sealer that is designed to initiate hydration using intrinsic moisture from dentin, with reported characteristics that include a clinically acceptable setting profile and radiopacity [20,21]. In addition to the difference in chemical base, AH Plus Bioceramic Sealer is supplied as a premixed hydraulic calcium silicate sealer, whereas AH Plus is supplied as a two-paste epoxy resin system. Accordingly, AH Plus Bioceramic differs from AH Plus not only in its setting mechanism, because it depends on moisture-driven hydration and early ion release rather than epoxy polymerization, but also in the clinical handling pathway required before placement [2,13,15,16,17,18,19,20,21]. Several investigations of calcium silicate-based sealers have also suggested improved sealing outcomes relative to conventional resin-based sealers, frequently attributed to hydration-related expansion and interfacial mineral precipitation [19,22,23]. At the same time, the hydration-dependent chemistry that underpins these potential advantages may increase sensitivity to chemical residues present immediately before or during early setting.

A central question is whether standard chelation protocols are fully compatible with the setting and early stability of hydraulic sealers when EDTA remnants remain. EDTA acts by chelating metal ions, including calcium, and can demineralize the inorganic phase of dentin [6,24]. Because calcium availability and ion exchange contribute to the hydration and maturation of tricalcium silicate-based materials [15,17,18], residual EDTA may plausibly interfere with early physicochemical behavior. Such interference could be reflected in increased solubility, altered alkalinizing potential (pH), reduced surface mechanical resistance, or changes in surface morphology and elemental composition, all of which are parameters linked to sealer stability at the dentin interface [3,25,26,27]. While epoxy resin sealers are generally regarded as chemically stable in aqueous conditions, direct comparative data on how brief exposure to residual 17% EDTA affects the physicochemical and surface characteristics of AH Plus Bioceramic Sealer in relation to AH Plus remain limited [2,13,20].

Accordingly, the aim of this in vitro study was to evaluate and compare the effect of exposure to 17% EDTA, used to simulate residual chelator after smear-layer removal, on early physicochemical and surface characteristics of AH Plus Bioceramic Sealer and AH Plus epoxy resin sealer. Solubility, eluate pH, Vickers microhardness, and surface morphology and elemental composition assessed by scanning electron microscopy and energy-dispersive X-ray spectroscopy (SEM/EDS) were analyzed after a 24 h evaluation period under standardized exposure conditions. The null hypothesis was that exposure to residual 17% EDTA would not induce significant changes in any of the evaluated properties for either material compared with the control exposure condition.

## 2. Materials and Methods

### 2.1. Study Design and Sample Preparation

This in vitro experimental study evaluated the effect of a brief pre-setting surface exposure to 17% EDTA on the physicochemical behavior and surface characteristics of two endodontic sealers: AH Plus Bioceramic (calcium silicate-based) and AH Plus (epoxy resin-based). Sample size was calculated using G*Power 3.1 (Heinrich-Heine-Universität Düsseldorf, Düsseldorf, Germany) assuming an effect size of 0.80, an α-error of 0.05, and a statistical power (1 − β) of 0.95, indicating that *n* = 12 specimens per experimental group were required for each quantitative endpoint. Accordingly, four experimental groups were established based on sealer type and pre-setting exposure solution: AHPB-DW, AHPB-EDTA, AHP-DW, and AHP-EDTA.

**Figure 1:**
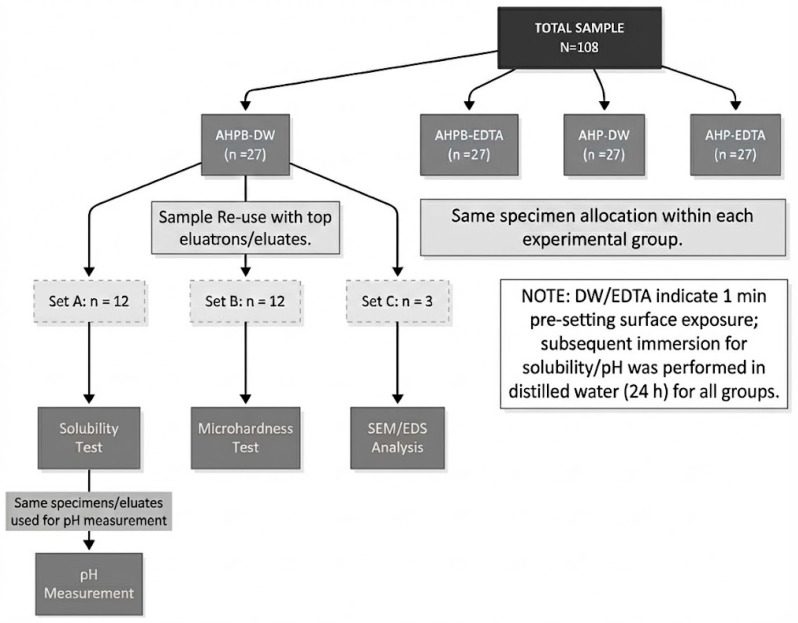
Specimen allocation and testing workflow (total *N* = 108; *n* = 27 per experimental group: AHPB-DW, AHPB-EDTA, AHP-DW, AHP-EDTA). Within each group, Subgroup A (*n* = 12) was used for solubility testing followed by eluate pH measurement using the same specimens/eluates; Subgroup B (*n* = 12) was used for Vickers microhardness testing; and Subgroup C (*n* = 3) was used for SEM/EDS. DW/EDTA indicate the 1 min pre-setting surface exposure condition; subsequent immersion for solubility/pH was performed in distilled water (24 h) for all Subgroup A specimens.

To accommodate independent specimen sets for different assays, 27 specimens were fabricated per experimental group (total *N* = 108) and allocated as follows: Subgroup A (*n* = 12/group; total *N* = 48) for solubility testing followed by eluate pH assessment using the same specimens/eluates; Subgroup B (*n* = 12/group; total *N* = 48) as an independent set for Vickers microhardness; and Subgroup C (*n* = 3/group; total *N* = 12) as an independent set for SEM/EDS surface characterization. Specimen allocation and the overall testing workflow are illustrated in Figure 1.

Standardized cylindrical molds (internal diameter 10.0 mm, height 1.5 mm) were designed using Fusion 360 (Autodesk, San Rafael, CA, USA) and fabricated via stereolithography using a dental 3D printer (NextDent 5100; NextDent, Soesterberg, The Netherlands) with a model resin (NextDent Model; NextDent, Soesterberg, The Netherlands). After printing, molds were washed in 99.9% isopropyl alcohol using a Wash and Cure unit (Anycubic Wash and Cure 3.0; Anycubic, Shenzhen, China), rinsed with distilled water, and post-cured for 10 min in a light-curing unit (LC-3D Print Box; NextDent, Soesterberg, The Netherlands) to ensure dimensional stability prior to specimen fabrication. The mold geometry included a base and circumferential walls that shielded the basal and lateral specimen surfaces while leaving the upper sealer surface unobstructed, enabling a controlled single-surface contact design for solution exposure and subsequent testing, consistent with a previously described methodology for standardized solution contact on unset sealer surfaces [26].

### 2.2. Specimen Exposure and Setting Protocol

**Figure 2:**
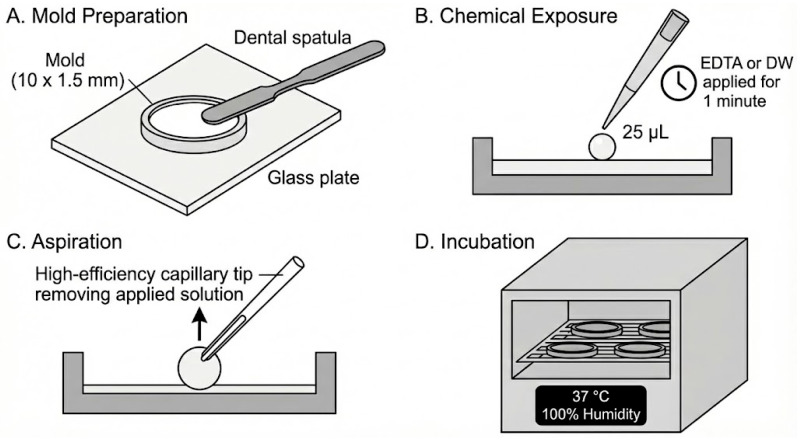
Workflow for mold preparation and pre-setting surface exposure of endodontic sealers to distilled water (DW) or 17% EDTA. (**A**) Mold preparation and specimen fabrication (10.0 mm diameter × 1.5 mm height). (**B**) Application of a 25 µL droplet of DW or 17% EDTA onto the unset sealer surface for 1 min. (**C**) Aspiration of the applied solution using a high-efficiency capillary tip. (**D**) Incubation at 37 °C and 100% relative humidity for the manufacturers’ setting time plus 50%.

Each sealer was dispensed into the molds according to the manufacturers’ instructions and gently compacted to minimize void formation. The upper surface was leveled flush with the mold margin. Specimens were allocated to one of two pre-setting exposure conditions: (i) distilled water (DW) or (ii) 17% EDTA. Using a calibrated micropipette (Accumax; Fine Care Biosystems, Gandhinagar, Gujarat, India), 25 µL of the assigned solution (17% EDTA; Zeyco, Zapopan, Mexico, or distilled water) was applied directly onto the exposed upper surface of the freshly placed sealer and maintained in contact for 1 min. The solution was then aspirated using high-efficiency capillary tips to simulate removal of residual irrigant/chelator prior to completion of obturation. This droplet-contact/aspiration approach (25 µL; 1 min) follows a previously established protocol for standardized short-term solution contact with unset sealer surfaces [26] (Figure 2).

After the exposure step, specimens were transferred to a humidified incubator (37 °C; 100% relative humidity) and maintained for a period corresponding to the manufacturers’ stated setting time plus 50% to ensure complete setting was achieved (6 h for AH Plus Bioceramic and 12 h for AH Plus). Importantly, EDTA was used only as a pre-setting surface exposure condition. Subgroup A specimens subsequently underwent the 24 h immersion protocol in distilled water for solubility testing and eluate pH assessment (using the same specimens/eluates), whereas Subgroup B and Subgroup C specimens were reserved exclusively for microhardness testing and SEM/EDS analysis, respectively (Figure 1).

### 2.3. Solubility Analysis

Solubility was determined using Subgroup A specimens (*n* = 12 per experimental group; total *N* = 48) according to ISO 6876:2012, with methodological adaptations to accommodate the 10 mm disc geometry and the single-surface exposure design. Glass vials were dehydrated and pre-weighed (m_0_) using an analytical balance (Adventurer; Ohaus, Parsippany, NJ, USA; readability 0.0001 g). After completion of the setting period, each specimen was weighed to obtain its initial mass (m_1_). Importantly, all Subgroup A specimens were immersed in distilled water for the solubility/pH assay, regardless of the pre-setting surface exposure solution (DW or 17% EDTA).

To preserve the single-surface contact model, each specimen was kept seated within its corresponding cylindrical mold during the 24 h immersion, so that only the upper surface (exposed circular area 78.5 mm^2^) was in contact with the immersion medium, while the basal and lateral surfaces remained shielded by the mold walls, consistent with previously described sealer–solution surface contact methodologies [26]. The mold-containing specimen was vertically positioned in 2.717 mL of distilled water in its respective vial. This volume was selected to maintain a surface-area-to-volume ratio of approximately 28.9 mm^2^/mL, calculated from the exposed circular surface area (78.5 mm^2^). Vials were sealed and incubated at 37 °C for 24 h.

After incubation, the mold-containing specimen was removed, and the exposed upper surface was rinsed with 2 mL of distilled water; the rinse was collected into the same vial to ensure complete recovery of eluates. The eluates were subsequently dehydrated in a vacuum drying oven at 110 °C until constant mass and the vials were reweighed (m_2_). Solubility (S) was calculated as:

S (%) = [(m_2_ − m_0_)/m_1_] × 100

where (m_2_ − m_0_) corresponds to the mass of the dried residue and m_1_ is the initial specimen mass.

### 2.4. Alkalizing Potential (pH)

The pH of the immersion medium (eluate) was measured for Subgroup A specimens after 24 h immersion in distilled water and prior to evaporation. Measurements were performed at 25 ± 1 °C using a digital pH meter (HI98103; Hanna Instruments, Woonsocket, RI, USA). The instrument was calibrated immediately before measurements using standard buffer solutions of pH 4, 7, and 14. The electrode was rinsed with distilled water and gently blotted dry between readings, and the pH value for each eluate was recorded directly from its corresponding vial.

### 2.5. Vickers Microhardness Testing

Vickers microhardness was evaluated using an independent specimen set (Subgroup B; *n* = 12 per experimental group; total *N* = 48). Although Subgroup B specimens were different from those used for solubility/pH (Subgroup A), they were fabricated using the same sample preparation and pre-setting surface exposure protocol described in Section 2.1 and Section 2.2 (standardized 10.0 mm × 1.5 mm discs prepared in the same molds; application of 25 µL of the assigned pre-setting surface exposure solution—distilled water (DW) or 17% EDTA—for 1 min onto the exposed upper surface, followed by aspiration; and incubation for the sealer-specific setting period). Subgroup B specimens were not subjected to the 24 h immersion procedure used for the solubility/pH workflow. After setting, specimens were removed from the molds and gently dried. The exposed upper surface was wet-polished using 1200-grit silicon carbide paper to obtain a standardized testing surface. Vickers microhardness was measured with a microhardness tester (Wilson VH1102; Buehler, Lake Bluff, IL, USA) under a 100 gf load applied for 10 s. Three indentations were performed per specimen in non-overlapping regions, and the mean Vickers hardness value (HV) was calculated for each specimen. All measurements were performed by a blinded operator.

### 2.6. Morphological and Chemical Characterization (SEM/EDS)

Surface morphology and elemental composition were assessed using a separate independent specimen set (Subgroup C; *n* = 3 per experimental group; total *N* = 12). As for Subgroups A and B, Subgroup C specimens were prepared using the same molds and the same sample preparation and pre-setting surface exposure/setting protocol described in Section 2.1 and Section 2.2 (standardized 10.0 mm × 1.5 mm discs; 25 µL of DW or 17% EDTA applied to the exposed upper surface for 1 min and subsequently aspirated; and incubation for the sealer-specific setting period). Subgroup C specimens were not subjected to the 24 h immersion procedure used for the solubility/pH workflow. After setting, specimens were removed from the molds, desiccated, and mounted on aluminum stubs using conductive adhesive tape, with analysis performed on the exposed upper surface. Surface topography was examined by scanning electron microscopy (TM4000Plus II; Hitachi, Tokyo, Japan) using secondary electron (SE) and backscattered electron (BSE) imaging at an accelerating voltage of 15 kV. Micrographs were acquired at magnifications consistent with those reported in the Results (e.g., ×100). Elemental composition was assessed by energy-dispersive X-ray spectroscopy (EDS) using the integrated detector at the same accelerating voltage (15 kV). EDS data were reported as semi-quantitative weight percentages (wt%) for the detected elements. These EDS measurements were used to identify relative surface trends and to complement the SEM observations, rather than to establish statistically tested elemental differences.

### 2.7. Statistical Analysis

Statistical analyses were performed using IBM SPSS Statistics for Windows (Version 24.0; IBM Corp., Armonk, NY, USA). Normality and homoscedasticity were assessed using the Shapiro–Wilk and Levene tests, respectively. Because solubility and eluate pH data did not meet parametric assumptions, comparisons among the four experimental groups were performed using the Kruskal–Wallis test followed by pairwise Mann–Whitney U tests with Bonferroni correction for multiple comparisons. Vickers microhardness was analyzed using one-way analysis of variance (ANOVA) followed by Tukey’s post hoc test. All tests were two-tailed, and the significance level was set at α = 0.05. For solubility/pH (Subgroup A) and microhardness (Subgroup B), inferential analyses were conducted with *n* = 12 specimens per experimental group. SEM/EDS outcomes (Subgroup C; *n* = 3 specimens per experimental group) were considered exploratory and were used to support morphological and elemental interpretation only. Because EDS provides semi-quantitative surface wt% data and this subset was not designed for inferential comparison, those results were reported descriptively and were not subjected to statistical hypothesis testing.

## 3. Results

**Figure 3:**
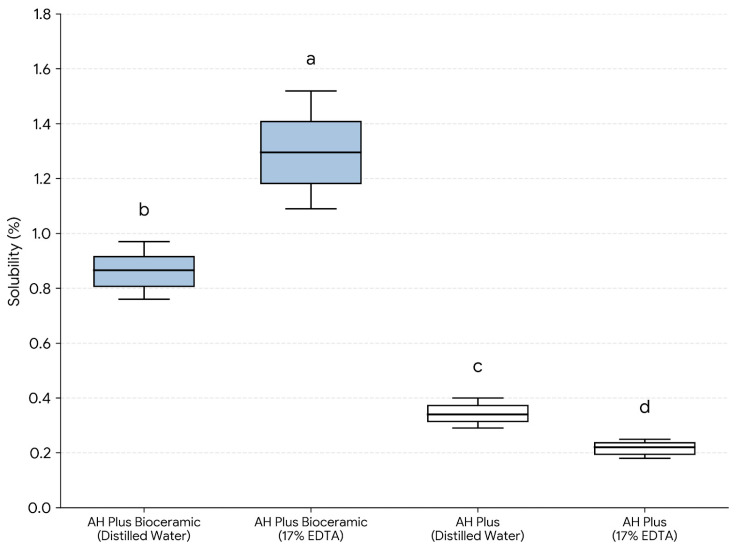
Solubility (%) of endodontic sealers after 24 h immersion in distilled water, according to the pre-setting surface exposure solution (distilled water or 17% EDTA) applied for 1 min prior to setting (*n* = 12/group). Different lowercase letters indicate statistically significant differences among groups (*p* < 0.05; Kruskal–Wallis test followed by pairwise Mann–Whitney U tests with Bonferroni adjustment).

**Figure 4:**
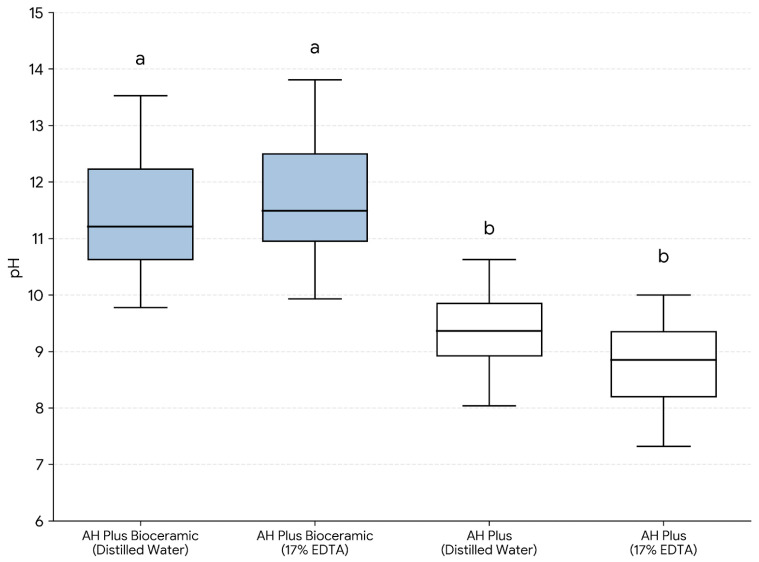
Eluate pH of endodontic sealers after 24 h immersion in distilled water, according to the pre-setting surface exposure solution (distilled water or 17% EDTA) applied to the unset material for 1 min prior to setting (*n* = 12/group). Different lowercase letters indicate statistically significant differences among groups (*p* < 0.05; Kruskal–Wallis test followed by pairwise Mann–Whitney U tests with Bonferroni adjustment).

**Figure 5:**
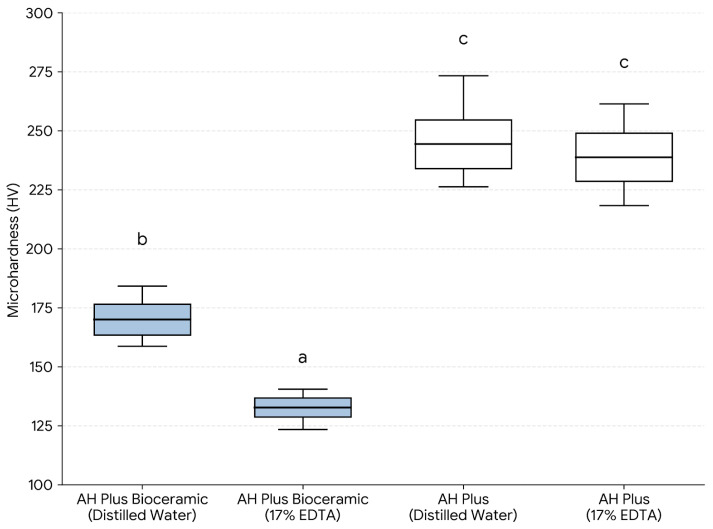
Vickers microhardness (HV) of endodontic sealers according to the pre-setting surface exposure solution (distilled water or 17% EDTA) (*n* = 12 per subgroup). Different lowercase letters indicate statistically significant differences among groups (*p* < 0.05; one-way ANOVA followed by Tukey’s post hoc test).

**Figure 6:**
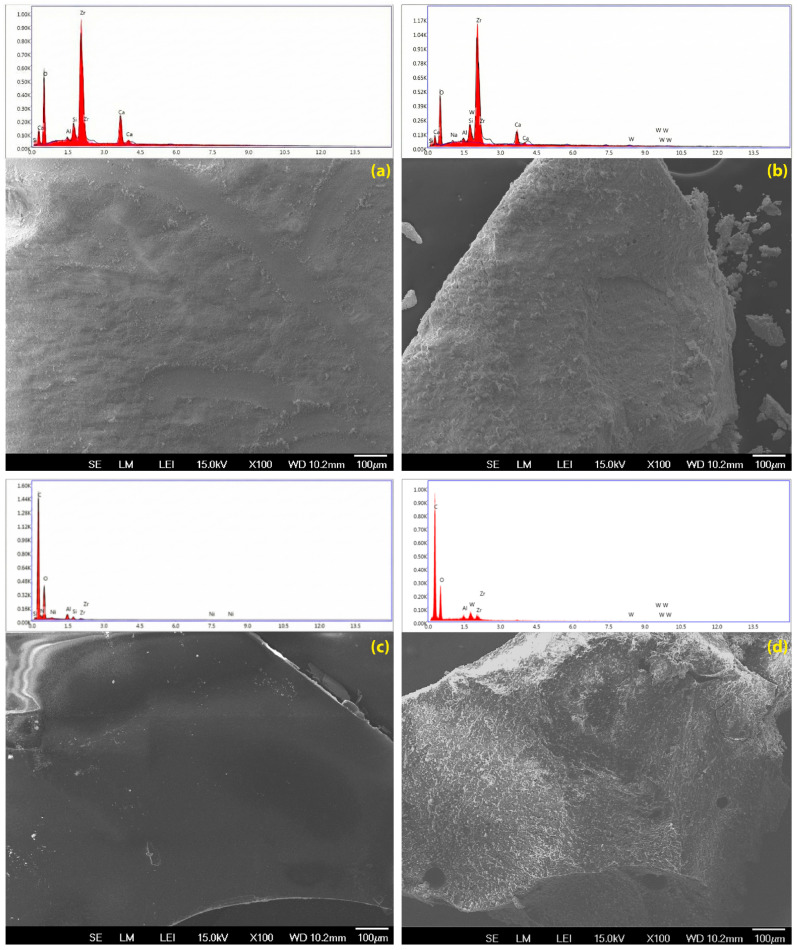
Representative SEM micrographs (100×) showing surface topography and their elemental analysis spectra. (**a**) AH Plus Bioceramic Sealer—distilled-water-exposed; (**b**) AH Plus Bioceramic Sealer—17% EDTA–exposed; (**c**) AH Plus—distilled-water-exposed; (**d**) AH Plus—17% EDTA-exposed.

The four experimental groups were defined by sealer type and the pre-setting surface exposure solution applied to the unset material (distilled water (DW) or 17% EDTA) prior to setting: AHPB-DW, AHPB-EDTA, AHP-DW, and AHP-EDTA. Solubility and eluate pH were assessed sequentially on the same specimens after 24 h immersion in distilled water (Table 1 and Table 2; Figure 3 and Figure 4). Vickers microhardness and SEM/EDS were evaluated using independent specimens prepared under the same pre-setting exposure conditions (Table 3 and Table 4; Figure 5 and Figure 6).

### 3.1. Solubility

Solubility values for Subgroup A specimens (*n* = 12/group) after 24 h immersion in distilled water are presented in Table 1 and Figure 3. In this manuscript, DW and 17% EDTA refer to the pre-setting surface exposure solution applied to the unset sealer for 1 min prior to setting. AH Plus Bioceramic Sealer exhibited higher solubility than AH Plus under both exposure conditions, with mean values of 0.86 ± 0.08% for AHPB–DW and 1.30 ± 0.16% for AHPB–EDTA, compared with 0.34 ± 0.04% for AHP–DW and 0.22 ± 0.03% for AHP–EDTA. The overall differences among the four groups were statistically significant (Kruskal–Wallis, *p* < 0.001). Post hoc pairwise comparisons (Mann–Whitney U tests with Bonferroni adjustment) indicated significant differences among groups (*p* < 0.05), as reflected by the distinct lowercase letters in Figure 3.

### 3.2. Eluate pH

Eluate pH values for Subgroup A specimens (*n* = 12/group) measured after 24 h immersion in distilled water are presented in Table 2 and Figure 4. In this manuscript, “distilled water” and “17% EDTA” indicate the pre-setting surface exposure solution applied to the unset sealer for 1 min prior to setting. AH Plus Bioceramic Sealer produced alkaline eluates, with mean pH values of 11.68 ± 1.54 (AHPB-DW) and 11.70 ± 1.39 (AHPB-EDTA), whereas AH Plus showed lower mean pH values of 9.30 ± 1.02 (AHP-DW) and 8.72 ± 1.00 (AHP-EDTA). Overall differences among the four groups were statistically significant (Kruskal–Wallis, *p* = 0.001). Post hoc pairwise comparisons (Mann–Whitney U tests with Bonferroni adjustment) indicated that both AH Plus Bioceramic subgroups had a significantly higher pH than both AH Plus subgroups (*p* < 0.05). Within each sealer, eluate pH did not differ significantly between DW and 17% EDTA pre-setting exposure after adjustment (*p* = 0.999 for AH Plus Bioceramic Sealer; *p* = 0.851 for AH Plus).

### 3.3. Vickers Microhardness

The Vickers microhardness values are summarized in Table 3 and Figure 5 (*n* = 12/group). Overall, AH Plus exhibited higher surface microhardness than AH Plus Bioceramic Sealer under both pre-setting exposure conditions. For AH Plus Bioceramic Sealer, mean microhardness decreased from 170.83 ± 9.76 HV in the distilled-water-exposed subgroup (AHPB-DW) to 131.72 ± 5.99 HV in the EDTA-exposed subgroup (AHPB-EDTA). For AH Plus, mean values were 246.92 ± 15.32 HV (AHP-DW) and 239.70 ± 15.10 HV (AHP-EDTA).

One-way ANOVA demonstrated significant differences among groups (F(3,44) = 249.52; *p* < 0.001). Tukey’s post hoc test indicated that AHPB-EDTA had significantly lower microhardness than AHPB-DW (*p* < 0.001), whereas no significant difference was detected between AHP-DW and AHP-EDTA (*p* = 0.475). Both AH Plus subgroups showed significantly higher microhardness than both AH Plus Bioceramic subgroups (*p* < 0.001).

### 3.4. Microscopic Inspection and Elemental Analysis (SEM/EDS)

Representative SEM micrographs (100×) are shown in Figure 6. The AH Plus Bioceramic Sealer surface in the distilled-water-exposed subgroup appeared relatively compact, with a granular texture and low apparent roughness (Figure 6a). In the EDTA-exposed subgroup, the bioceramic surface exhibited more pronounced topographical irregularities, with heterogeneous regions and loosely adherent particulate debris, particularly at the specimen periphery (Figure 6b).

In the AH Plus distilled-water-exposed subgroup, the surface appeared predominantly smooth and homogeneous, with limited localized imperfections (Figure 6c). Following EDTA exposure, AH Plus displayed a roughened appearance with increased surface pitting while maintaining an overall continuous matrix (Figure 6d).

Semi-quantitative EDS findings are provided in Table 4 and should be interpreted descriptively as relative surface trends. For AH Plus Bioceramic Sealer, the dominant detected elements were Zr and O, with Ca detected at 12.68 wt% in the distilled-water-exposed subgroup (AHPB-DW). In the EDTA-exposed subgroup (AHPB-EDTA), the Ca signal was lower (7.31 wt%) and the relative Zr signal was higher (42.61 to 51.74 wt%). These descriptive differences should not be interpreted as an absolute increase in zirconium, but rather as a relative change in the elemental profile. For AH Plus, the elemental profile remained dominated by C (~69 wt%) and O (~22 wt%), with W (~6–7 wt%) detected as a radiopacifier and only minor descriptive changes between exposure conditions.

## 4. Discussion

This study evaluated whether pre-setting surface exposure to 17% EDTA, used to simulate potential chelator remnants at obturation, influences the early physicochemical behavior and surface characteristics of a hydraulic calcium silicate sealer (AH Plus Bioceramic Sealer) compared with an epoxy resin sealer (AH Plus). The principal finding was that sealer chemistry was the dominant determinant of 24 h behavior, whereas EDTA exposure produced a material-dependent response that was most evident for the hydraulic sealer in surface-related outcomes. Clinically, this distinction is relevant because the sealer–dentin interface is created immediately after irrigation, at a time when residual chelator may still persist within canal irregularities or re-emerge from dentinal tubules [8,9,10,11,12].

The 1 min exposure protocol was adapted from the single-surface contact model described by Kapralos et al. [26], in which unset sealer specimens were protected on the basal and lateral surfaces and challenged only on the exposed upper surface with a standardized liquid volume before aspiration. That model is useful because complete canal dryness is not always achieved clinically, particularly in the apical third and in anatomically complex systems. Accordingly, the present design was intended to reproduce a short and clinically plausible contact between freshly placed sealer and residual solution at the onset of setting, rather than prolonged immersion.

This open-surface design should therefore be interpreted as a conservative or worst-case surface challenge. In the clinical root canal, the sealer comes into contact not only with residual fluid but also dentin, which may partially buffer chelator activity, adsorb irrigant remnants, and provide a calcium-containing substrate that could attenuate or modify early surface reactions, particularly in hydraulic calcium silicate sealers. Accordingly, the present model was intended to isolate the direct effect of residual EDTA on the exposed sealer surface rather than to fully reproduce the complexity of the sealer–dentin interface.

At 24 h, AH Plus Bioceramic Sealer exhibited higher solubility than AH Plus under both exposure conditions. This pattern is consistent with previous studies reporting that AH Plus Bioceramic and other calcium silicate sealers display greater solubility and ion release than AH Plus, which reflects their more reactive hydration chemistry [15,16,17,18,19,20]. By contrast, epoxy resin sealers typically show lower solubility because polymerization yields a dense crosslinked network with limited fluid sorption and fewer leachable fractions [2,13].

Within each material, pre-setting EDTA exposure significantly modified solubility, increasing it for AH Plus Bioceramic Sealer and decreasing it for AH Plus. For the calcium silicate-based sealer, the increase was compatible with calcium chelation during early hydration, which may have increased the leachable fraction and is coherent with the reduction in surface calcium detected by EDS [6,24]. In the broader literature, Jose et al. [28] reported that commonly used irrigants, including 17% EDTA, altered setting-related behavior and metal composition in sealers of different chemical bases, supporting the concept that freshly mixed sealers are sensitive to irrigant remnants. Although their study evaluated different endpoints and did not include AH Plus Bioceramic, our findings complement that evidence by showing that even brief pre-setting surface contact with EDTA can materially affect the early behavior of a hydraulic sealer.

Both AH Plus Bioceramic subgroups generated markedly alkaline eluates and significantly exceeded AH Plus. This is expected for calcium silicate sealers, which release calcium and hydroxyl ions during hydration, thereby increasing alkalinity and contributing to their reported bioactive potential [15,16,17,18,19,20,21]. In contrast, epoxy resin sealers generally show pH values closer to neutrality because their setting is polymer-based rather than dependent on hydroxyl ion release [2,13].

Notably, pre-setting EDTA exposure did not significantly alter eluate pH within either sealer at 24 h. Because EDTA chelates calcium, a reduction in alkalinity could be anticipated for hydraulic materials [6,24]. Under the present model, however, the short exposure time and subsequent 24 h immersion in distilled water may have limited any measurable bulk effect. Therefore, the absence of a pH difference should not be interpreted as absence of interaction; rather, it suggests that brief EDTA contact can leave bulk alkalinizing behavior largely unchanged while still producing detectable surface modifications.

Microhardness provides an indirect assessment of surface mechanical resistance and is commonly used as a surrogate indicator of early setting and surface maturation in endodontic sealers [25,26,27]. In the present study, AH Plus exhibited markedly higher microhardness than AH Plus Bioceramic Sealer under both exposure conditions, which is consistent with the rigid crosslinked matrix expected after epoxy polymerization [2,13]. Within AH Plus, pre-setting EDTA exposure did not significantly affect microhardness, suggesting that the short exposure-and-aspiration protocol did not materially impair the early surface integrity of the epoxy resin sealer.

In contrast, AH Plus Bioceramic Sealer showed a significant reduction in microhardness after EDTA exposure. Because EDTA chelates calcium [6,24], brief contact at the material surface before setting may reduce local calcium availability and/or remove calcium-rich early hydration products, thereby impairing near-surface hydration and early maturation of the calcium silicate hydrate matrix [15,17,18]. This interpretation is strengthened by the concomitant SEM/EDS findings. It also parallels the broader concept reported by Kapralos et al. [26], who showed that brief pre-setting chemical exposure can alter physicochemical properties of unset sealers, even when the contact is standardized and short.

SEM observations supported a material-dependent response to EDTA exposure. AH Plus maintained a relatively continuous surface matrix and showed only mild roughening and pitting after EDTA exposure, which is consistent with its low solubility and chemical stability [2,13]. By contrast, the bioceramic sealer showed more evident topographical irregularity and loosely attached particulate debris after EDTA. The accompanying EDS data revealed a marked reduction in surface calcium and a relative increase in zirconium signal, supporting a mechanism of calcium depletion and surface phase redistribution. Because EDS data were expressed as semi-quantitative relative wt%, the higher zirconium signal should not be interpreted as an absolute increase in zirconium content. Rather, it likely reflects an apparent relative enrichment of the less soluble zirconium-containing phase after calcium depletion and/or preferential loss of more soluble surface constituents during early EDTA-mediated surface degradation.

These surface findings are relevant when compared directly with previous literature. Jose et al. [28] reported that exposure to commonly used irrigants, including 17% EDTA, altered setting-related behavior and metal composition in endodontic sealers of different chemical bases. Although their study focused on setting time and metal composition rather than solubility, microhardness, and early surface morphology, both studies converge in showing that freshly mixed sealers are sensitive to irrigant remnants. Our results extend that concept by showing that, under a brief pre-setting surface contact model, AH Plus Bioceramic Sealer was more susceptible than AH Plus, particularly in microhardness reduction and calcium-related surface changes.

Siddique et al. [29] investigated precipitate formation after interaction among endodontic irrigants rather than sealer properties directly. For that reason, their work is not comparable at the level of physicochemical sealer outcomes; however, it is still relevant because it demonstrates that residual irrigants and their reaction products can modify the chemical environment present immediately before obturation. In that sense, their findings support the clinical premise of the present study: even when bulk pH remained unchanged at 24 h, residual solution chemistry could still influence the early surface condition of a hydraulic calcium silicate sealer.

Taken together, the present data indicate that residual EDTA is more critical for the early behavior of the hydraulic calcium silicate sealer than for the epoxy resin sealer under the tested conditions. Therefore, the null hypothesis was rejected for solubility and for surface-related outcomes in AH Plus Bioceramic Sealer, whereas it was not rejected for eluate pH within either material at 24 h.

## 5. Limitations

Several limitations should be considered. First, this in vitro model evaluated a brief pre-setting surface exposure followed by early testing and therefore does not fully reproduce the clinical complexity of chelator retention within irregular canal anatomy or the sealer–dentin interface; accordingly, it may represent a conservative worst-case surface challenge rather than the full clinical environment. Second, solubility and pH were assessed at a single 24 h time point, so later maturation-related effects may have been missed. Moreover, because the adapted solubility method relied on evaporation of the immersion medium and recovery of dried residue, the calculated residue mass may have been influenced by factors that were not separately quantified in the present design, including hygroscopic behavior of dried residues, possible carryover of residual EDTA-derived salts after the pre-setting exposure/aspiration step, and loss of volatile components during drying. Accordingly, solubility values should be interpreted primarily as comparative estimates obtained under standardized conditions rather than as absolute measurements of material dissolution. Third, SEM/EDS findings were derived from an exploratory subset (*n* = 3 per subgroup) and were semi-quantitative; accordingly, they should be interpreted as corroborative relative surface trends rather than statistically tested elemental differences or absolute bulk composition. Finally, the study did not evaluate bond strength, sealer penetration, or long-term leakage; therefore, the present outcomes should be interpreted as early surface and physicochemical responses rather than direct predictors of long-term sealing performance.

## 6. Conclusions

Within the limitations of this in vitro study, sealer chemistry was the primary determinant of early behavior after 24 h. AH Plus Bioceramic Sealer exhibited higher solubility and markedly higher alkalinizing potential than AH Plus, whereas AH Plus showed higher surface microhardness under both pre-setting exposure conditions. Brief pre-setting surface contact with 17% EDTA produced material-dependent effects: it increased solubility and significantly reduced surface microhardness in AH Plus Bioceramic Sealer, while eluate pH remained unchanged within each material at 24 h. Descriptive SEM/EDS findings also suggested that EDTA exposure was associated with greater surface irregularity and lower surface calcium in the bioceramic sealer, whereas AH Plus showed comparatively limited surface and elemental variation. Clinically, these findings support careful final irrigation, adequate canal drying, and thorough removal of chelating residues before obturation, particularly when hydraulic calcium silicate sealers are used. Further studies should evaluate longer aging periods and dentin-sealer interfacial outcomes under clinically representative irrigation sequences.

## Figures and Tables

**Table 1:** Solubility (%) after 24 h immersion in distilled water by pre-setting surface exposure solution (distilled water or 17% EDTA; 1 min) for AH Plus Bioceramic Sealer and AH Plus (epoxy resin) (*n* = 12 per subgroup). Data are mean ± SD, median, and min–max.

Sealer Group	Pre-Setting Exposure Solution	Mean (%) ± SD	Median	Min–Max
AH Plus Bioceramic	Distilled Water	0.86 ± 0.08	0.87	0.76–0.97
	17% EDTA	1.30 ± 0.16	1.3	1.09–1.52
AH Plus (Resin)	Distilled Water	0.34 ± 0.04	0.34	0.29–0.40
	17% EDTA	0.22 ± 0.03	0.22	0.18–0.25

**Table 2:** Eluate pH after 24 h immersion in distilled water by pre-setting surface exposure solution (distilled water or 17% EDTA; 1 min) for AH Plus Bioceramic Sealer and AH Plus (epoxy resin) (*n* = 12 per subgroup). Data are mean ± SD, median, and min–max.

Sealer Group	Pre-Setting Exposure Solution	Mean pH ± SD	Median	Min–Max
AH Plus Bioceramic	Distilled Water	11.68 ± 1.54	11.21	9.78–13.53
	17% EDTA	11.70 ± 1.39	11.49	9.93–13.81
AH Plus (Resin)	Distilled Water	9.30 ± 1.02	9.36	8.04–10.63
	17% EDTA	8.72 ± 1.00	8.85	7.32–10.00

**Table 3:** Vickers microhardness (HV) by pre-setting surface exposure solution (distilled water or 17% EDTA; 1 min) for AH Plus Bioceramic Sealer and AH Plus (epoxy resin) (*n* = 12 per subgroup). Data are mean ± SD, median, and min–max.

Sealer Group	Pre-Setting Exposure Solution	Mean (HV) ± SD	Median	Min–Max
AH Plus Bioceramic	Distilled Water	170.83 ± 9.76	169.95	158.7–184.2
	17% EDTA	131.72 ± 5.99	132.7	123.4–140.5
AH Plus (Resin)	Distilled Water	246.92 ± 15.32	244.3	226.3–273.3
	17% EDTA	239.70 ± 15.10	238.75	218.3–261.4

**Table 4:** Semi-quantitative EDS elemental composition (wt%) of sealer surfaces according to the pre-setting surface exposure solution (distilled water or 17% EDTA; 1 min) applied to the unset material prior to setting. Values are presented descriptively for the exploratory SEM/EDS subset (*n* = 3 per subgroup) to illustrate relative surface trends only; they were not subjected to inferential statistical analysis and should not be interpreted as absolute bulk composition. ND—not detected.

Sealer	Pre-Setting Exposure Solution	C	O	Al	Si	Ca	Fe	Zr	W
AH Plus Bioceramic	Distilled Water	1.13	40.74	0.56	0.88	12.68	0.15	42.61	ND
	17% EDTA	1.64	37.98	0.13	0.97	7.31	0.14	51.74	ND
AH Plus	Distilled Water	68.67	22.47	ND	ND	0.73	ND	0.89	6.96
	17% EDTA	69.12	22.77	ND	ND	0.8	ND	0.68	6.28

## Data Availability

The original contributions presented in this study are included in the article. Further inquiries can be directed to the corresponding author.
